# Groundwater Chemistry and Children's Blood Lead Levels: A County‐Wise Analysis in the United States

**DOI:** 10.1029/2025GH001670

**Published:** 2026-01-16

**Authors:** Emily V. Pickering, Xianqiang Fu, Rajesh Melaram, Farhad Jazaei, Alasdair Cohen, Debra Bartelli, Chunrong Jia, Hongmei Zhang, Xichen Mou, Abu Mohd Naser

**Affiliations:** ^1^ Division of Epidemiology, Biostatistics and Environmental Health School of Public Health University of Memphis Memphis TN USA; ^2^ Department of Civil Engineering The University of Memphis Memphis TN USA; ^3^ Department of Population Health Sciences Virginia Tech Blacksburg VA USA

**Keywords:** water corrosiveness, blood lead level, groundwater chemistry, lead leaching

## Abstract

Groundwater is a major source of drinking water in the United States (US). Groundwater chemistry can contribute to lead leaching from water supply pipes due to factors such as pH and mineral content that influence corrosion. Lead exposure disproportionately affects children from low‐income neighborhoods. We evaluated the association of county‐level groundwater chemicals with the percentage of children with blood lead levels >5 μg/dL (BLL5%) in 1,104 US counties served by public water utilities using groundwater. Out of the 4,844 BLL5% observations, 3,525 had values of “NA” for BLL5%. We used weighted least squares regression to evaluate the associations, adjusting for covariates such as county‐level median household income, educational attainment, and poverty rates. Bayesian Kernel Machine Regression (BKMR) was used to assess the joint effects of all chemicals on BLL5%. Sensitivity analyses tested the robustness of our results by imputing missing BLL5% values. A one mg/L increase in arsenic, copper, dissolved oxygen, and selenium was associated with increases in BLL5% of 0.0512% (95% CI: 0.0002%, 0.1023%), 0.0358% (95% CI: 0.0208%, 0.0508%), 0.0956% (95% CI: 0.0225%, 0.1687%), and 0.3038% (95% CI: 0.1747%, 0.4420%), respectively. Alkalinity, pH, calcium, bicarbonate, and dissolved solids were not found to be statistically significant. BKMR identified calcium, lithium, and alkalinity (posterior inclusion probabilities = 1,000) as important, though with minimal effects. Sensitivity analyses showed variability in results depending on assumptions about missing data. Our findings highlight the importance of monitoring groundwater quality and implementing interventions to reduce childhood lead exposure risks in vulnerable populations, particularly minority, and low‐income children.

## Plain Language Summary

Certain groundwater qualities, such as pH and mineral content, can cause pipes to corrode, leading to higher lead levels in drinking water, especially in areas with older pipes. Lead is highly toxic to children and can cause learning and behavioral issues. Our research found that groundwater chemicals like arsenic, copper, dissolved oxygen, and selenium were linked to more children with harmful blood lead levels. However, chemicals like alkalinity and calcium, which may reduce lead, showed mixed results. Future studies should examine individual diets and blood lead levels to better understand these risks.

## Key Points

- Groundwater arsenic, copper, dissolved oxygen, and selenium linked to higher county‐level childhood blood lead levels
- Protective indicators like pH, alkalinity, and calcium showed inconsistent associations with blood lead
- Results highlight the need for groundwater monitoring and targeted interventions to reduce lead exposure

## 1 Introduction

Lead was widely used in late 19th‐century United States (US) infrastructure, including in distribution pipes and paint (Dignam et al., 2019). Negative health outcomes of lead exposure have been documented since the 19th century (Riva et al., 2012). Yet, the US did not ban the use of lead paint until 1978 (United States Environmental Protection Agency, 2024c) or the use of lead in the creation and repair of plumbing for water consumption until 1986 (United States Environmental Protection Agency, 1989). After these bans, lead pipes already constructed stayed in use, and there are still an estimated 9.2 million lead distribution pipes in the US (Epa & Mohotti, 2016). Lead exposure through drinking water largely occurs from lead pipe corrosion due to certain water characteristics, including low pH and low mineral content, causing lead to enter the drinking water (United States Environmental Protection Agency, 2024a). The EPA instated the Lead and Copper Rule, which requires customer pipes to be monitored, and action must be taken to stop corrosion if lead concentration exceeds an action level of 15 μg/L (United States Environmental Protection Agency, 2023, 2024b). In October 2024, this rule was revised to require the replacement of the large majority of lead pipes within 10 years and reduced the action level to 10 μg/L (United States Environmental Protection Agency, 2024a). Although federal funding supports lead service line replacement, current funding is insufficient to meet national needs, raising concerns about timely implementation. With lead pipes still in use, there is a risk of lead exposure from water distribution systems.

Approximately 590,000 children in the US between the ages of 1 and 6 had blood lead levels greater than 5 µg/dL in 2016 (Jacobs & Brown, 2023). Children are more at risk for lead exposure for many reasons, including ingesting more lead and absorbing more lead through their gastrointestinal tract than adults (Abelsohn & Sanborn, 2010). Childhood lead exposure can lead to significant cognitive, behavioral, and physical impairments, including lower IQs, poor motor skills, poor memory, symptoms of attention deficit hyperactivity disorder, decreased adult brain volume, and brain damage in severe cases (Abelsohn & Sanborn, 2010; Vorvolakos et al., 2016). Lead exposure is never safe and is dangerous even in very small amounts; cognitive impairments, including lower IQ, have been observed in children with blood lead levels below 5 µg/dL (Mayans, 2019).

Lead exposure also disproportionately affects minority and low‐income communities (Leech et al., 2016). These individuals are more likely to live in neighborhoods with houses built before the ban on lead paint and plumbing, putting them at higher risk for lead exposure (Karp, 2023). The highest risks for lead exposure were found to be living in low‐income housing, followed by being black (Yeter et al., 2020). In fact, non‐Hispanic black children were found to have 2.8 times the likelihood of having blood lead levels greater than 5 µg/dL compared to their white peers (Yeter et al., 2020). Children in 13% of US households using private groundwater wells face a 25% higher risk of elevated blood lead levels (Gibson et al., 2020). This increased risk is likely due to private wells not being regulated under the Safe Drinking Water Act; without proper testing and treatment by users, the water can contain higher lead levels (United States Environmental Protection Agency, 1974).

Our study aims to evaluate the relationship between groundwater chemicals and the burden of county‐wise childhood lead exposure in the US. Certain water quality indicators, such as low pH and low concentration of groundwater metals (e.g., calcium, magnesium, iron, sodium), can increase the corrosiveness of water and can lead to increased lead intake if lead pipes are corroded. While previous studies have demonstrated the overarching effects of groundwater chemistry on lead leaching and exposure, there remains a critical gap in understanding how localized groundwater conditions interact with socioeconomic factors to influence lead exposure levels in children. By understanding what groundwater chemicals are associated with an increase in children with lead exposure and which are most important in explaining this relationship, future public health interventions could be implemented in communities disproportionately affected by groundwater‐chemistry‐impacted lead exposure in drinking water.

## 2 Materials and Methods

### 2.1 Data Source and Processing

We obtained the county‐wise water source data from the Water Fluoridation Reporting System within the CDC's Oral Health Data System, which reports county‐level water source types for public water systems (Centers for Disease Control and Prevention, 2024a, 2024b). We restricted our analyses to counties that relied on at least one groundwater source for their public water supply by the water utility companies and excluded counties reporting the use of only surface water sources by water utility companies. The publicly available county‐wise lead exposure data on children <72 months was obtained from the CDC's Childhood Lead State Surveillance Data, with years ranging from 2012 to 2017 (Centers for Disease Control and Prevention, 2024a, 2024b). The groundwater quality parameters data were obtained from the Geochemical Database for the Brackish Groundwater Assessment of the US, collected by the US Geological Survey, which includes a wide range of chemical data on 124,000 groundwater wells compiled from 16 sources across the US and its territories, ranging from the years 1901–2013 (United States Geological Survey, 2024). Our study was restricted to only the continental US and focused on 30 groundwater chemicals. Although groundwater composition may be modified during municipal treatment and distribution, source‐water properties remain a primary determinant of corrosion potential. Our focus was therefore on groundwater chemistry as an upstream driver of corrosivity and potential lead mobilization from pipes. The county‐level covariates were sourced from the American Community Survey (United States Census Bureau, 2025) and included 2021 data for each county on unemployment rate, median household income, percent of adults with less than a high school education, and percent of children aged 0 to 17 in poverty. This data was then merged into the previously combined data sets.

### 2.2 Statistical Analysis

**Figure 1:**
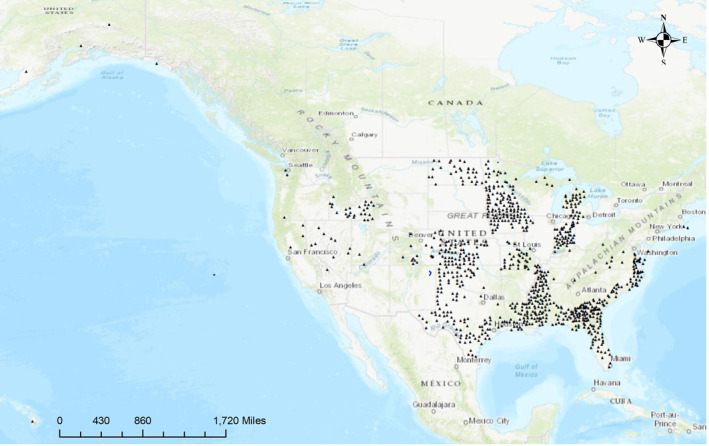
Location of United States counties used in analyses.

Thirty groundwater chemicals were analyzed in 1,104 counties in 22 US states (Figure 1). We calculated the median and mean values for all chemicals across each county. We generated boxplots to show each groundwater chemical's distribution, variability, and potential outliers. These measurements reflect source‐water chemistry; tap‐water data were not available. Many counties had more than 1 year of BLL data. A total of 4,844 BLL measurements were included in the analyses. Out of the 4,844 observations, 3,525 had values of “NA” for the number of children with blood lead levels greater than 5 μg/dL (BLL5%), indicating instances where there were six or fewer children with BLL5 in a county. These values were intentionally hidden by the CDC to protect confidentiality. We excluded the counties with “NA” values for BLL5 from the main analysis and imputed them for sensitivity analysis. We used weighted least squares (WLS) regression models to explore the relationship between county‐wise median values of each chemical and the percentage of children with BLL5%. We implemented separate models for each groundwater chemical, and all models were weighted for the total number of children in each county and adjusted for county‐level covariates: unemployment rate, median household income, percent of adults with less than a high school education, and percent of children aged 0 to 17 in poverty. Forest plots were created to illustrate a visual summary of the effects of groundwater chemicals on BLL5%, highlighting both the estimated size and the 95% confidence intervals.

To estimate the joint effects of all groundwater chemicals on county‐wise BLL5% and to evaluate which chemicals influenced BLL5% the most, we implemented Bayesian Kernel Machine Regression (BKMR) with a Gaussian kernel function, while adjusting for county‐level covariates. Model fitting used 10,000 iterations of Markov Chain Monte Carlo sampling. The BKMR model can be written as:

$$Y_{i}=h\left(z_{i1,}z_{i2,}\text{…},z_{i30}\right)+X_{i}\beta +\epsilon_{i}$$

where ${\;Y}_{i}$ represents the BLL5% for observation *i*, *h*(*z*) represents the joint effects of the groundwater characteristics, $z_{i1,}z_{i2,}\text{…},z_{i30}$, $X_{i}\beta$ represents the linear effects of the county‐level covariates, and $\epsilon_{i}$ represents residual error. Component‐wise variable selection was accomplished using posterior inclusion probabilities (PIPs), where a higher PIP indicates a chemical strongly influenced county‐wise BLL5%. Single variable risk summaries and their 95% confidence intervals were used to estimate the individual contribution of each groundwater characteristic to the outcome. These summaries estimated the change in BLL5% associated with shifting each groundwater characteristic from the 25th to the 75th quantile while keeping the rest of the predictors constant at their median value.

As part of sensitivity analyses, we implemented WLS and BKMR regressions to explore the relationship between county‐wise median and mean values of each chemical and BLL5% after replacing “NA” values to zero, randomly set to 1 through 5, and 6, separately. All data processing and statistical analysis were performed using RStudio (R Core Team, 2024) and the suites of *R* packages employed in this work include “dplyr,” “tidyr,” “bkmr,” “gt,” and “ggplot2.”

## 3 Results

**Figure 2:**
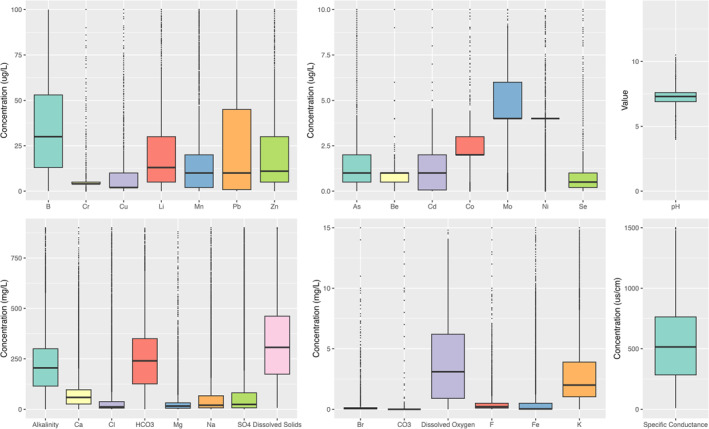
Box plots demonstrating distribution of groundwater ions or characteristics in all selected counties.

The median and interquartile ranges (IQR) for selected chemicals were as follows: pH, 7.3 (IQR: 6.9–7.6); alkalinity, 206 mg/L (IQR: 115–301); HCO_3_, 243 mg/L (IQR: 128–354); dissolved oxygen, 3.1 mg/L (IQR: 0.9–6.2); total dissolved solids, 360 mg/L (IQR: 199–642); selenium, 0.5 μg/L (IQR: 0.2–1); copper, 2 μg/L (IQR:2–10); and arsenic, 1 mg/L (IQR: 0.5–2) (Figure 2).

**Figure 3:**
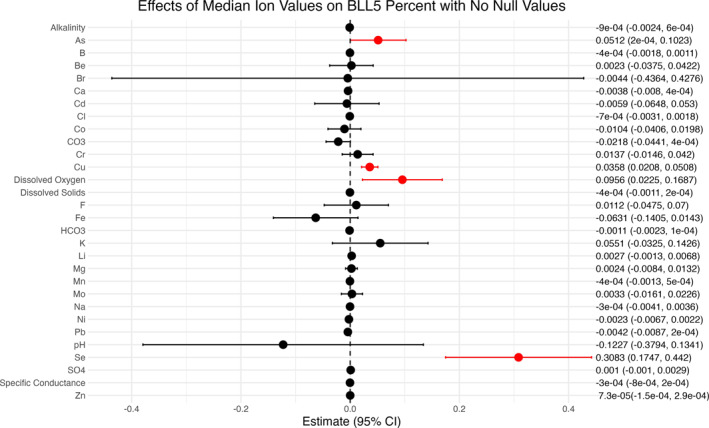
Association between one unit increase in median of groundwater chemicals and change in % of children with elevated blood lead levels above 5 μg/dl. The significance level was determined using a *p* value less than 0.05.

WLS regression resulted in the following significant associations between county‐wise groundwater chemicals and BLLs. Each one mg/L increase in median arsenic was associated with a 0.0512% increase in BLL5% (95% CI: 0.0002, 0.1023). A one mg/L increase in median copper was linked to a 0.0358% increase in BLL5% (95% CI: 0.0208, 0.0508). A one mg/L increase in median dissolved oxygen was associated with a 0.0956% increase in BLL5% (95% CI: 0.0225, 0.1687). A one μg/L increase in median selenium led to a 0.30383% increase in BLL5% (95% CI: 0.1747, 0.4420) (Figure 3). The following ions were not found to be significant in our analysis, but have been previously found to have significant associations with lead exposure. A one‐unit increase in median pH was linked to a 0.1227% decrease in BLL5% (95% CI: −0.3794, 0.1341). A one mg/L increase in alkalinity corresponded to a $9\times 10^{-4}\%$ decrease in BLL5% (95% CI: −0.0024, $6\times 10^{-4}$). A one mg/L increase in median calcium was associated with a 0.0038% decrease (95% CI: −0.008, 0.0004), and a one mg/L increase in iron was associated with a 0.0631% decrease (95% CI: −0.1405, 0.0143) in BLL5%. An increase of one mg/L in bicarbonate showed a 0.0011% decrease in BLL5% (95% CI: −0.0023, 0.0001), and a one mg/L increase in dissolved solids was linked to a $4\times 10^{-4}\%$ BLL5% decrease (95% CI: −0.0011, $2\times 10^{-4}$) (Figure 3). Groundwater lead was not significantly associated with BLL5% in our WLS models. Other statistically non‐significant ions were boron, beryllium, bromide, cadmium, chloride, cobalt, copper, chromium, carbonate (CO_3_), fluoride, potassium, lithium, magnesium, manganese, molybdenum, sodium, nickel, pH, sulfate (SO_4_^2−^), specific conductance, and zinc. When the county‐level mean chemical exposure was considered, higher groundwater calcium, iron, and lead concentrations were associated with statistically significant decreases in BLL5% (Figure S1 in Supporting Information S1).

From BKMR, calcium, lithium, and alkalinity had high posterior inclusion probabilities (PIP = 1,000), indicating strong inclusion in the model. However, their effect estimates on BLL5% were minimal, with calcium showing −0.0196% (95% CI: −0.2690, 0.2299), lithium showing −0.0142% (95% CI: −0.1609, 0.1894), and alkalinity showing −0.0089% (95% CI: −0.2821, 0.2644). Selenium had a PIP of 0.7182 with an estimate of −0.0001% (95% CI: −0.0251, 0.0254) (Table 1).

**Table 1:** Bayesian Kernel Machine Regression Results When Null Values of BBL5% Are Excluded

Variable	PIP	Estimate
Ca	1.0000	−0.0196 (−0.2690, 0.2299)
Li	1.0000	0.0142 (−0.1609, 0.1894)
Alkalinity	1.0000	−0.0089 (−0.2821, 0.2644)
Se	0.7182	0.0001 (−0.0251, 0.0254)
F	0.2742	0.0000 (−0.0065, 0.0066)
As	0.2384	0.0000 (−0.0089, 0.0090)
Br	0.1632	0.0000 (−0.0013, 0.0013)
Fe	0.1284	0.0000 (−0.0073, 0.0074)
K	0.1116	0.0003 (−0.0188, 0.0193)
pH	0.1068	−0.0001 (−0.0155, 0.0153)
Mg	0.0878	0.0012 (−0.1764, 0.1788)
CO_3_	0.0854	0 (0, 0)
Cd	0.0450	−0.0000 (−0.0069, 0.0068)
Mo	0.0320	0.0006 (−0.0615, 0.0626)
Be	0.0216	−0.0000 (−0.0034, 0.0033)
Co	0.0048	−0.0000 (−0.0057, 0.0057)
Cu	0.0046	0.0001 (−0.0180, 0.0182)
Zn	0.0000	0 (0, 0)
Specific conductance	0.0000	0 (0, 0)
Dissolved oxygen	0.0000	0 (0, 0)
Dissolved solids	0.0000	0 (0, 0)
Na	0.0000	0 (0, 0)
HCO_3_	0.0000	0 (0, 0)
SO_4_	0.0000	0 (0, 0)
Cl	0.0000	0 (0, 0)
B	0.0000	0 (0, 0)
Cr	0.0000	0 (0, 0)
Pb	0.0000	0 (0, 0)
Mn	0.0000	0 (0, 0)
Ni	0.0000	0 (0, 0)

The sensitivity analyses revealed inconsistent results for the ions that were statistically significant in the main analyses, while the findings for the nonsignificant ions remained relatively consistent across analyses (Figures S1–S7 in Supporting Information S1). When “NA” values were replaced by a random number between 1 and 5, each one unit increase in median pH was associated with a 0.1444% (95% CI: −0.2623, −0.0265) decrease in BLL5%, a one mg/L increase in alkalinity was linked to a $7\times 10^{-4}$% (95% CI: −0.0013, 0) decrease in BLL5%, a one mg/L increase in calcium was associated with a $5\times 10^{-4}$% (95% CI: −0.0016, $6\times 10^{-4}$) decrease in BLL5%, a one mg/L increase in bicarbonate was associated with a $7\times 10^{-4}$% (95% CI: −0.0013, $-1\times 10^{-4}$) decrease in BLL5%, and 1 mg/L increase in dissolved solids was linked to a $3\times 10^{-5}$% (95% CI: $-2.9\times 10^{-4}$, $-2.2\times 10^{-4}$) decrease in BLL5% (Figure S2 in Supporting Information S1). Groundwater iron and lead concentrations were associated with a reduction of BLL5% in most sensitivity analyses (Figures S2–S7 in Supporting Information S1). Sensitivity analyses using BKMR suggested inconsistent results in terms of the influence of chemicals on BLL5% (Tables S2–S4 in Supporting Information S1). Most chemicals had negligible effects, and their credible intervals crossed zero, indicating no statistical significance (Tables S2–S4 in Supporting Information S1).

## 4 Discussion

This study integrates data from multiple sources to evaluate the relationship between groundwater chemical concentrations and the prevalence of elevated blood lead levels in children across 1,104 counties in 22 US states relying on groundwater. The results demonstrated significant associations between arsenic, copper, dissolved oxygen, and selenium and BLL5%, but these findings were inconsistent across different statistical approaches. The variability in results for significant ions highlights the sensitivity of statistical findings to methodological decisions, such as the approach to missing data imputation. These discrepancies suggest that some observed associations may be context‐dependent and should be interpreted cautiously.

Several groundwater constituents that showed positive associations with BLL5% in our models may influence lead exposure through various indirect pathways. For example, higher dissolved oxygen can increase water corrosivity by promoting oxidative reactions that destabilize protective pipe scales, including lead carbonate and mixed iron–lead deposits, thereby facilitating greater lead release into drinking water (Agency, 2016; Nguyen et al., 2010). Copper can also amplify lead release through galvanic corrosion, which occurs when copper and lead components are electrically connected within premise plumbing. Controlled laboratory studies demonstrate that copper‐lead galvanic pairs create acidic, chloride‐rich microenvironments at the lead surface, substantially accelerating lead dissolution compared to lead alone. Arsenic and selenium, although not themselves corrosive agents in drinking water systems, frequently occur in groundwater characterized by low alkalinity, low hardness, and higher redox potential, which are hydrogeochemical conditions known to promote lead solubility (Ayotte et al., 2011; Smedley & Kinniburgh, 2002).

Although previous studies have suggested that higher alkalinity (Tam & Elefsiniotis, 2009), pH (Tam & Elefsiniotis, 2009), bicarbonate (Levin, 1986), and calcium (Syofyan et al., 2020) levels in groundwater may have protective effects against elevated blood lead levels by reducing lead leaching and bioavailability, our analyses did not consistently support this evidence. While WLS regression showed minor protective effects for bicarbonate, alkalinity, and pH, with reductions in BLL5%, these associations were not significant or consistent in our BKMR models. Several factors may explain why our findings were not entirely consistent with prior studies that have shown protective effects of alkalinity, pH, calcium, and bicarbonate. First, although our groundwater data reflect raw source‐water chemistry, public water utilities routinely modify and condition groundwater before it enters the distribution system, including pH adjustment, softening, aeration, and the addition of corrosion‐control agents such as orthophosphate. These treatment steps substantially alter parameters such as pH, alkalinity, and calcium content, meaning that the chemistry ultimately governing lead corrosion at the tap may differ markedly from the original groundwater composition. Second, the groundwater chemistry data span multiple decades and characterize long‐term geochemical conditions rather than the contemporaneous water quality that children were exposed to, limiting temporal alignment between exposure and outcome. Third, ecological heterogeneity, including variation in pipe materials, distribution system age, corrosion‐control practices, and household plumbing, may attenuate or obscure the protective associations identified in prior controlled studies.

Low pH in groundwater can result from natural processes and environmental pollutants, such as emissions from power plants, vehicles, and agricultural activities, which leach into water sources (United States Environmental Protection Agency, 2025). Acidic water increases corrosivity, especially in areas with lead pipes, accelerating corrosion and lead leaching into drinking water (Organization, 2007). In contrast, higher pH levels promote the formation of a corrosion scale in lead pipes, consisting of protective compounds like cerussite and plumbonacrite, which reduce lead leaching (Kim & Herrera, 2010; Nordberg et al., 1985; Organization, 2007). Utility companies enhance this protection by adding phosphate ions, which react with lead to form an insoluble layer within the pipes (Pieper et al., 2019). However, acidic water destabilizes this scale, exposing the lead pipe to water and increasing lead leaching.

Like low pH, low dissolved solids contribute to pipe corrosion and lead leaching into drinking water (Benham et al., 2011). Higher total dissolved solids, particularly higher concentrations of calcium and calcium carbonate, add to the creation of a corrosion scale in distribution pipes. Thus, without these minerals in groundwater, there will likely not be a substantial enough protective layer between the water and lead pipes, contributing to the oxidation of lead and lead leaching.

Lead ingestion can be more dangerous for those lacking certain essential minerals (Talpur et al., 2018). For example, low calcium intake is associated with higher blood lead levels, likely due to these compounds using the same biological mechanisms to be absorbed into the gastrointestinal track (Kordas, 2017). Calcium and lead compete for access to gastrointestinal transporters and without calcium to reduce lead's access to these transporters, the body will intake more lead (Kordas, 2017). Higher lead absorption levels have been found in individuals with low vitamin C, iron, and zinc (Talpur et al., 2018). Yet, calcium was neither significant in our WLS analyses nor identified as a key contributor in BKMR. This discrepancy could stem from differences in groundwater composition across study regions, limitations in county‐level data, or methodological differences in our statistical approaches. These findings suggest that the protective effects of these parameters may not be universally applicable and require further investigation in diverse settings.

Higher groundwater iron levels were consistently associated with lower county‐level prevalence of elevated blood lead levels. Elevated groundwater iron may support the development of iron‐oxide scales that adsorb or incorporate lead into the pipe‐scale matrix, thereby limiting dissolved Pb release (Bae et al., 2020; Trueman et al., 2017). In addition, biological mechanisms are also relevant. Iron deficiency increases intestinal expression of divalent metal transporter‐1 (DMT1) (Kayaaltı et al., 2015), which facilitates absorption of both iron and lead; thus, adequate iron status may reduce gastrointestinal lead uptake. Our findings reinforce both chemical and physiological pathways through which iron may mitigate lead exposure.

The inverse associations observed for groundwater lead and BLL5% in several sensitivity analyses was similar to the findings for groundwater iron. In many systems, water utilities remove or condition groundwater before distribution through aeration, filtration, or corrosion‐control treatment in a way that elevated groundwater lead does not necessarily translate into elevated tap‐water lead. Under the Lead and Copper Rule, water utilities monitor lead through tap‐water sampling in high‐risk homes rather than routine testing of source groundwater, as lead in drinking water primarily arises from corrosion of lead‐containing distribution and premise‐plumbing materials (Devine & Triantafyllidou, 2023; Emmnett Environmental Law & Clinic, 2025). Nevertheless, source‐water quality assessments conducted for treatment design or well management often guide decisions about pH and alkalinity adjustment, orthophosphate dosing, aeration, filtration, or blending, which stabilize water chemistry and minimize lead release from distribution pipes and plumbing (Bradley & Horscroft, 2018; Wasserstrom et al., 2017). This is consistent with our observation that counties with higher groundwater lead levels had lower BLL5%.

Minority and low‐income households are more likely to be affected by the corrosion of lead distribution pipes, as they are more likely to live in houses serviced by lead distribution pipes (Gochfeld & Burger, 2011). While public water supplies are supposed to be monitored for lead concentrations, lead and copper rule violations do occur. In 2015, there were lead copper rule violations from 5,363 water systems that served over 18 million individuals (Pieper et al., 2019), further increasing the risk of lead exposure in these high‐risk communities. Even in those low‐income minority communities where the lead copper rule is enforced, the possibility of ineffective corrosion control methods leading to low‐dose lead exposure is a dangerous possibility (Pieper et al., 2019). Those who use private well water as their primary drinking water source are also more affected by lead exposure (Gibson et al., 2020). The Environmental Protection Agency does not regulate private wells and thus are not covered by the Safe Drinking Water Act 37; in fact, 12%–19% of private wells in the US were found to exceed the lead action draw level of 15 μg/L, putting them at high risk of lead exposure (Pieper et al., 2015).

In our analyses, the nonsignificant ions showed more consistent findings across statistical approaches. For example, ions like boron, bromide, calcium, and chloride consistently exhibited low PIPs and effect estimates close to zero, regardless of the analytical method or how missing data were treated. This consistency reinforces the conclusion that these ions likely have minimal or no impact on BLL5%.

Our study had several limitations. This was an ecological analysis of county‐wise data, suggesting that these results cannot be interpreted at an individual level. We did not have individual‐level data on the water sources people are consuming, meaning children in our analysis could be primarily drinking surface or bottled water. The childhood lead data had a high number of null values for children's blood lead levels, making analysis difficult; this is likely due to the common practice of excluding values less than six for privacy. Dietary intake of essential minerals (e.g., calcium) is also important when exploring blood lead levels, and this information was not included in our analysis. Also, the CDC's Childhood Lead State Surveillance Data used a blood lead reference value of 5 mg/dL, but the CDC recently changed the blood lead reference level to 3.5 mg/dL (Ruckart, 2021). Finally, groundwater chemistry may not precisely represent tap‐water chemistry, as treatment processes at the utility level (e.g., corrosion control, pH adjustment, orthophosphate addition) can substantially modify water corrosivity and metal solubility.

This study identified significant associations between groundwater chemicals, such as arsenic, copper, dissolved oxygen, and selenium, and elevated blood lead levels (BLL5%) in children and raised questions as to the assumed protective effects of chemicals like bicarbonate and calcium, and physicochemical parameters like alkalinity and pH, for reducing the likelihood of corrosion and lead leaching. The findings underscore the importance of targeted groundwater quality monitoring, especially in vulnerable communities such as those living in older homes and those who use private wells, to mitigate lead exposure risks. Future research should prioritize children's longitudinal individual‐level data, including drinking water ion and dietary mineral intake and blood lead concentrations, for the causal effect of groundwater ions on lead exposure.

## Conflict of Interest

The authors declare no conflicts of interest relevant to this study.

## Supporting information

Supporting Information S1

## Data Availability

All data reported in this study are preserved and available publicly from the US federal agencies. The major ions data is available from the USGS (United States Geological Survey, 2024). County‐level lead data is available from the CDC (Centers for Disease Control and Prevention, 2024a, 2024b).
