# Supplementary material for: Groundwater Chemistry and Children's Blood Lead Levels: A County‐Wise Analysis in the United States
Source: Geohealth. 2026 Jan 16;10(1):e2025GH001670. doi: 10.1029/2025GH001670 (PMC12809049; doi:10.1029/2025GH001670)

*GeoHealth*

Supporting Information for

**Groundwater chemistry and children’s blood lead levels: A county-wise analysis in the United States**

Emily V. Pickering^1^, Xianqiang Fu^1^, Rajesh Melaram^1^, Farhad Jazaei^2^, Alasdair Cohen^3^, Debra Bartelli^1^, Chunrong Jia^1^, Hongmei Zhang^1^, Xichen Mou^1^, Abu Mohd Naser^1^

Authors’ affiliation:

^1^ Division of Epidemiology, Biostatistics and Environmental Health, School of Public Health, University of Memphis, Memphis, TN

^2^Department of Civil Engineering, The University of Memphis, Memphis, TN

^3^Department of Population Health Sciences, Virginia Tech, Blacksburg, VA

**Contents of this file**

Figure S1: Association between one unit increase in mean of groundwater chemicals and change in % of children with elevated blood lead levels above 5 µg/dl. The significance level was determined using a p-value less than 0.05.

Figure S2: Association between one unit increase in median of groundwater chemicals and change in % of children with elevated blood lead levels above 5 µg/dl with NA values of BLL5 set to 0. The significance level was determined using a p-value less than 0.05.

Figure S3: Association between one unit increase in mean of groundwater chemicals and change in % of children with elevated blood lead levels above 5 µg/dl with NA values of BLL5 set to 0. The significance level was determined using a p-value less than 0.05.

Figure S4: Association between one unit increase in median of groundwater chemicals and change in % of children with elevated blood lead levels above 5 µg/dl with NA values of BLL5 set randomly to 1-5. The significance level was determined using a p-value less than 0.05.

Figure S5: Association between one unit increase in mean of groundwater chemicals and change in % of children with elevated blood lead levels above 5 µg/dl with NA values of BLL5 set randomly to 1-5. The significance level was determined using a p-value less than 0.05.

Figure S7: Association between one unit increase in mean of groundwater chemicals and change in % of children with elevated blood lead levels above 5 µg/dl with NA values of BLL5 set to 6. The significance level was determined using a p-value less than 0.05.

Table S1: List of groundwater chemicals used in analyses.

Table S2: Results of BKMR using median values of groundwater chemicals when setting null BLL5 to 0, randomly to 1-5, and 6.

Table S3: Results of BKMR using mean values of groundwater chemicals when null values of BLL5 were excluded and set to 0.

Table S4: Results of BKMR using mean values of groundwater chemicals when setting null BLL5 randomly to 1-5 and to 6.

Table S1: List of groundwater chemicals used in analyses

| **Measures** | **Formula** | **Unit** |
| --- | --- | --- |
| pH |  |  |
| Dissolved oxygen |  | mg/L |
| Dissolved solids |  | mg/L |
| Specific conductance |  | µs/cm |
| Beryllium | Be | µg/L |
| Calcium | Ca | mg/L |
| Magnesium | Mg | mg/L |
| Lithium | Li | µg/L |
| Potassium | K | mg/L |
| Sodium | Na | mg/L |
| Bicarbonate | HCO_3_^-^ | mg/L |
| Carbonate | CO_3_^-^ | mg/L |
| Sulfate | SO_4_^2-^ | mg/L |
| Bromide | Br^-^ | mg/L |
| Chloride | Cl^-^ | mg/L |
| Fluoride | F^-^ | mg/L |
| Boron | B | µg/L |
| Calcium Carbonate | CaCO_3_ | mg/L |
| Selenium | Se | µg/L |
| Arsenic | As | µg/L |
| Cadmium | Cd | µg/L |
| Chromium | Cr | µg/L |
| Cobalt | Co | µg/L |
| Copper | Cu | µg/L |
| Iron | Fe | mg/L |
| Lead | Pb | µg/L |
| Manganese | Mn | µg/L |
| Molybdenum | Mo | µg/L |
| Nickel | Ni | µg/L |
| Zinc | Zn | µg/L |


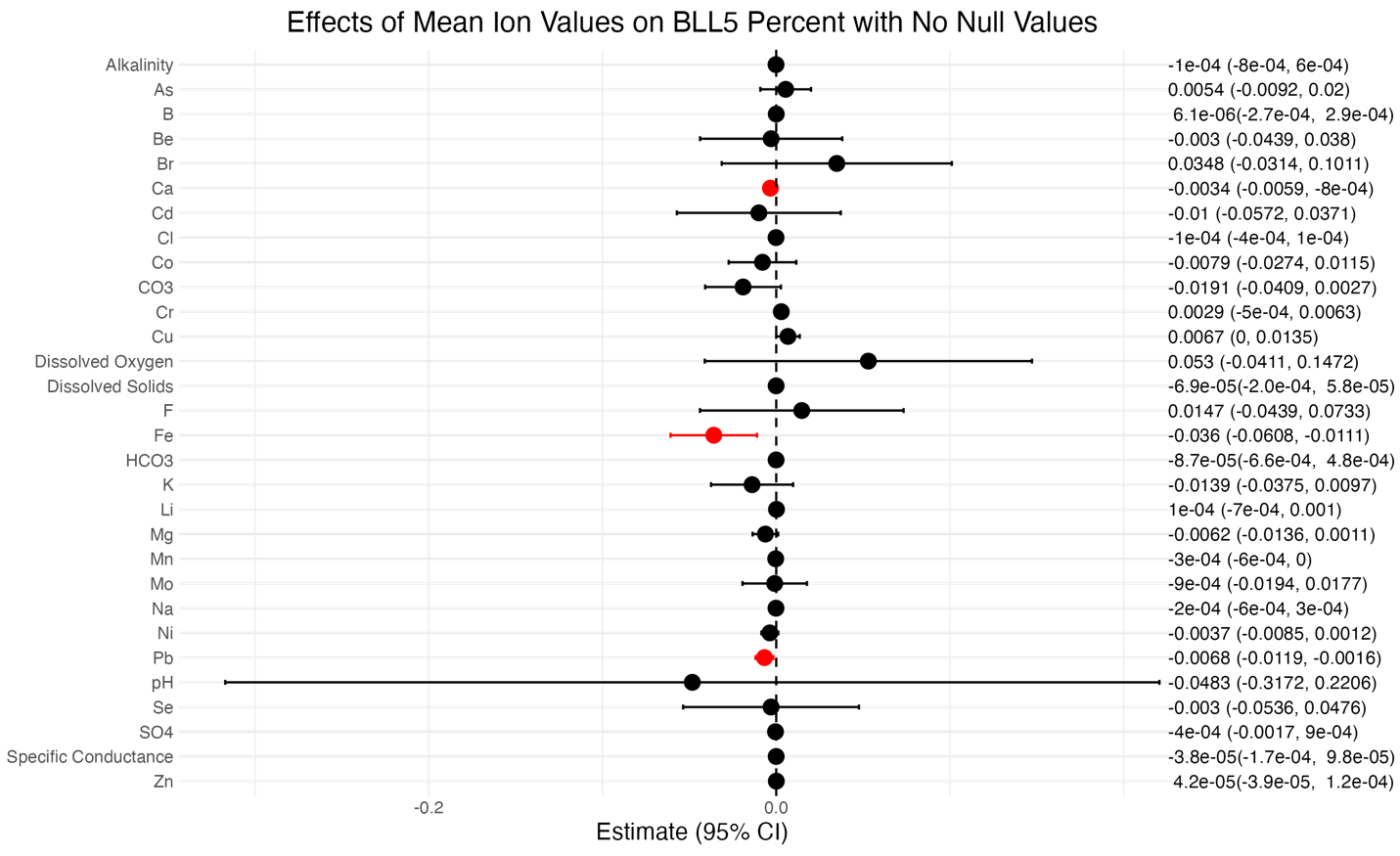
Figure S1: Association between one unit increase in mean of groundwater chemicals and change in % of children with elevated blood lead levels above 5 µg/dl. The significance level was determined using a p-value less than 0.05.


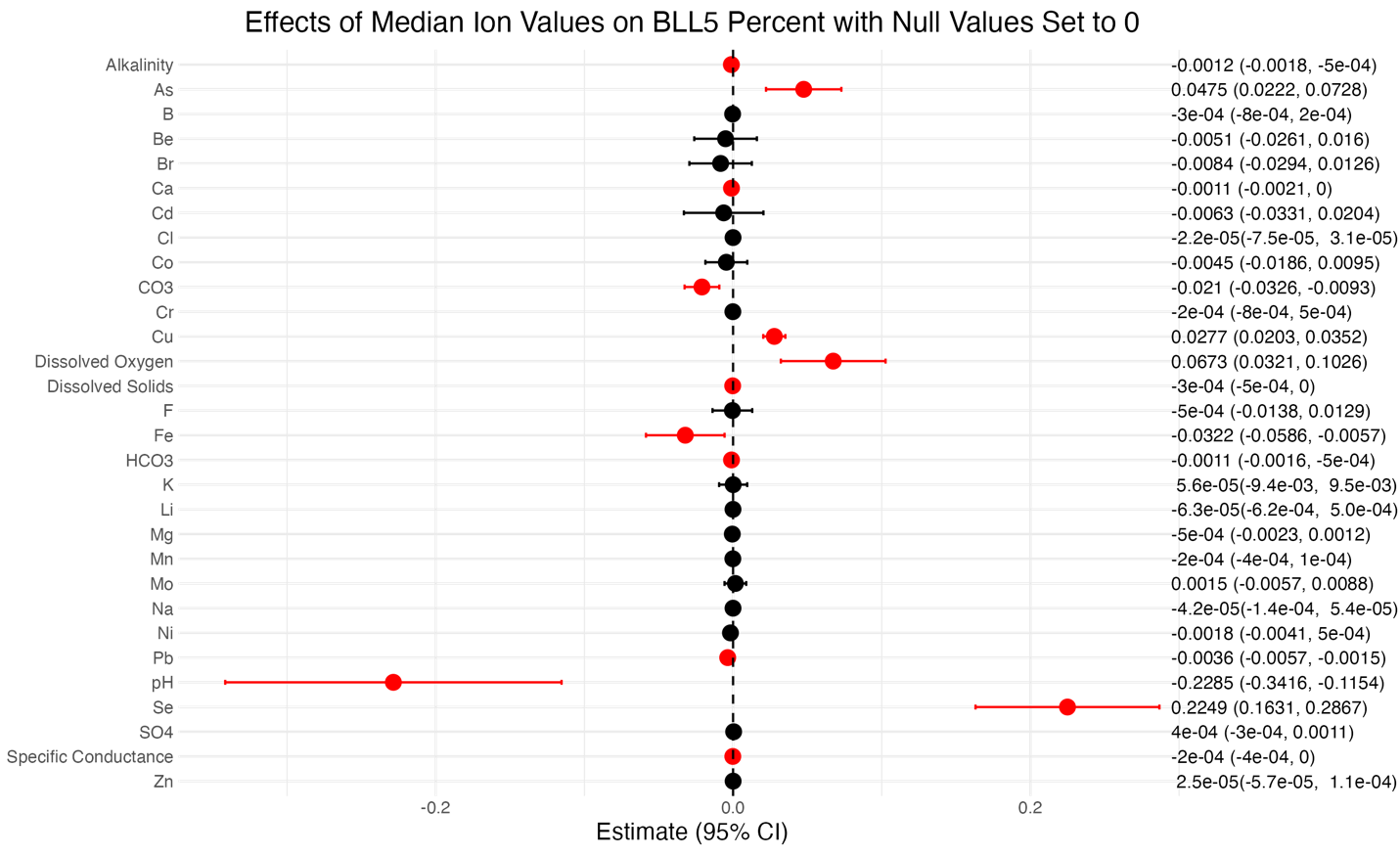


Figure S2: Association between one unit increase in median of groundwater chemicals and change in % of children with elevated blood lead levels above 5 µg/dl with NA values of BLL5 set to 0. The significance level was determined using a p-value less than 0.05.


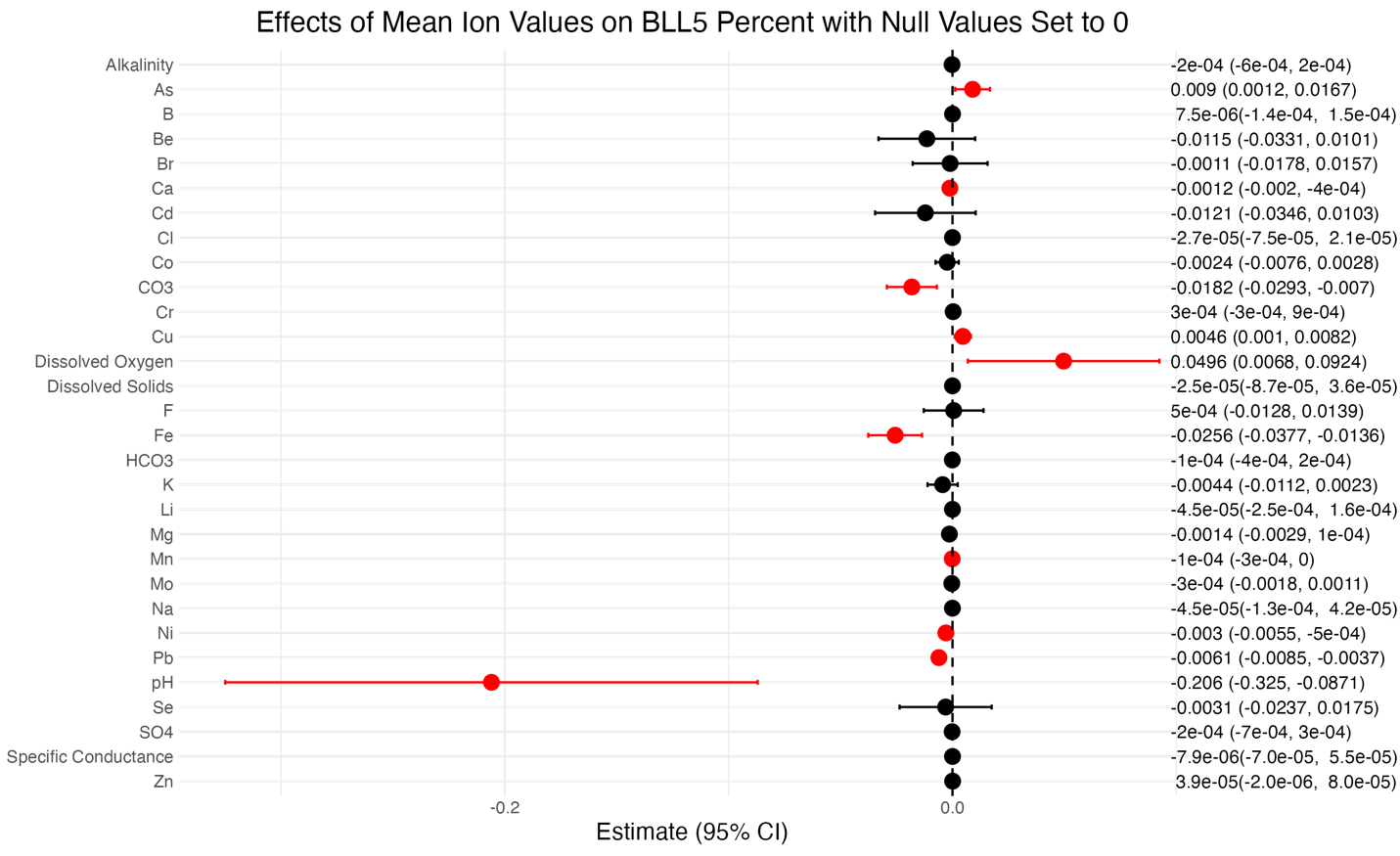


Figure S3: Association between one unit increase in mean of groundwater chemicals and change in % of children with elevated blood lead levels above 5 µg/dl with NA values of BLL5 set to 0. The significance level was determined using a p-value less than 0.05.


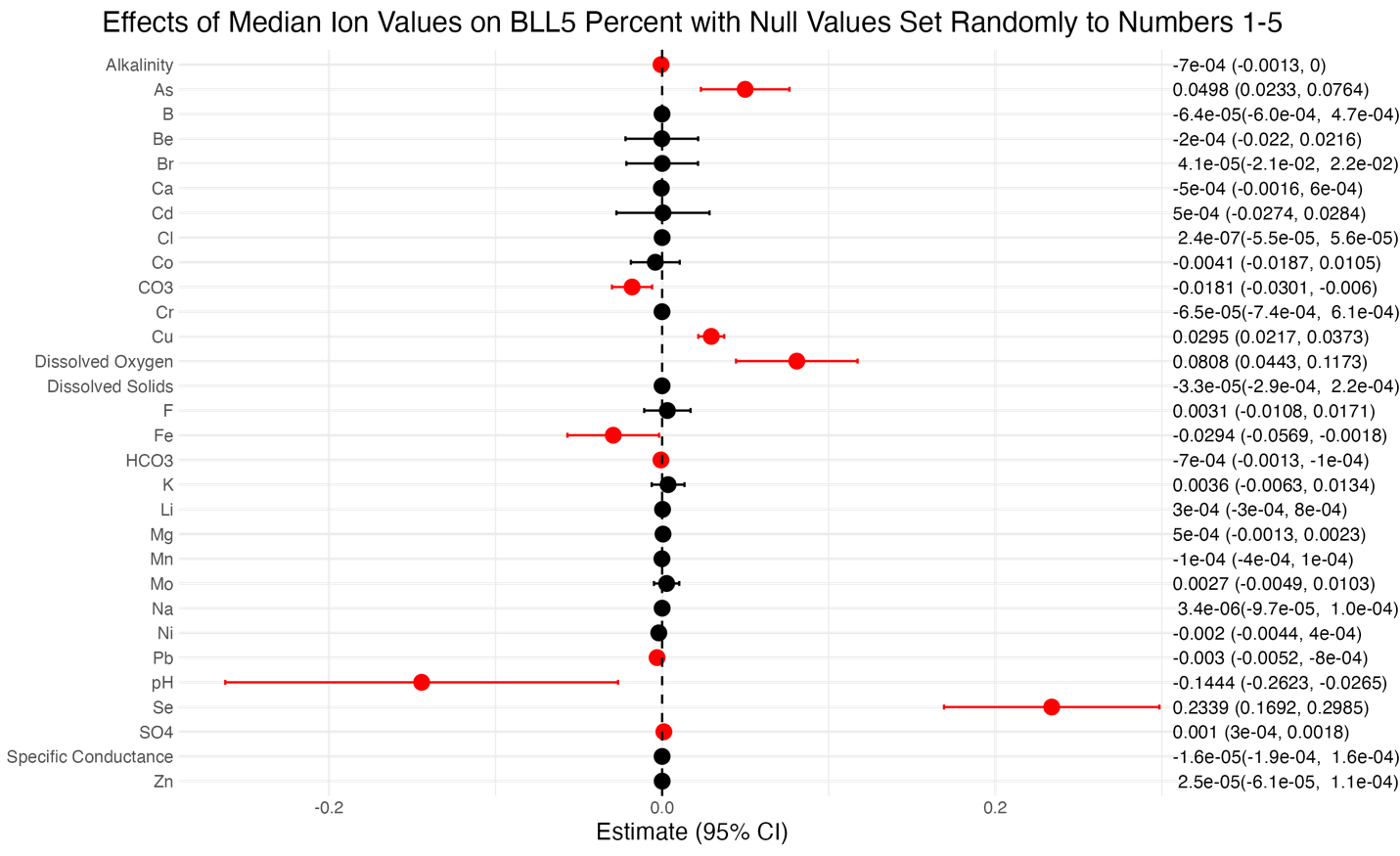


Figure S4: Association between one unit increase in median of groundwater chemicals and change in % of children with elevated blood lead levels above 5 µg/dl with NA values of BLL5 set randomly to 1-5. The significance level was determined using a p-value less than 0.05.


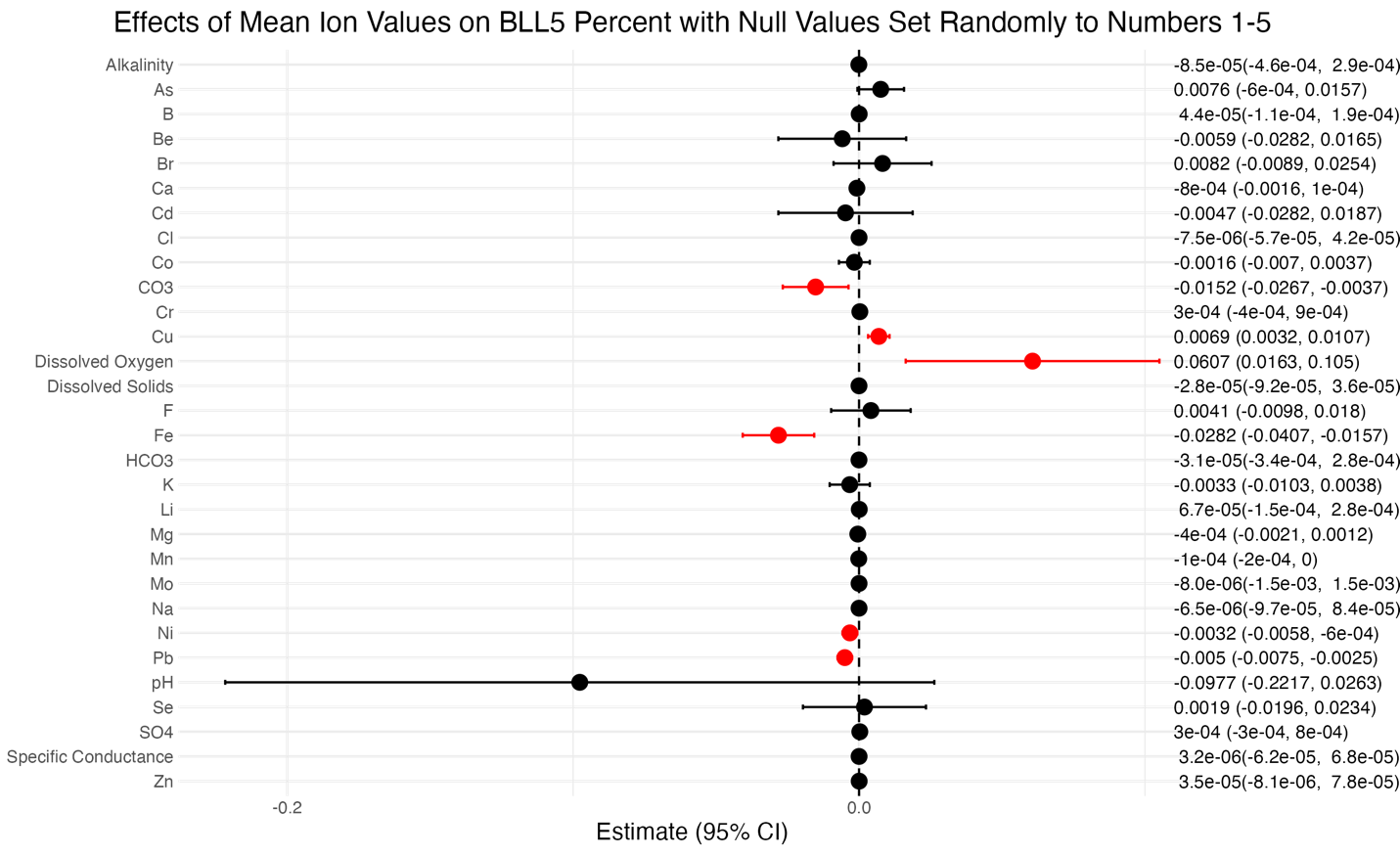
Figure S5: Association between one unit increase in mean of groundwater chemicals and change in % of children with elevated blood lead levels above 5 µg/dl with NA values of BLL5 set randomly to 1-5. The significance level was determined using a p-value less than 0.05.


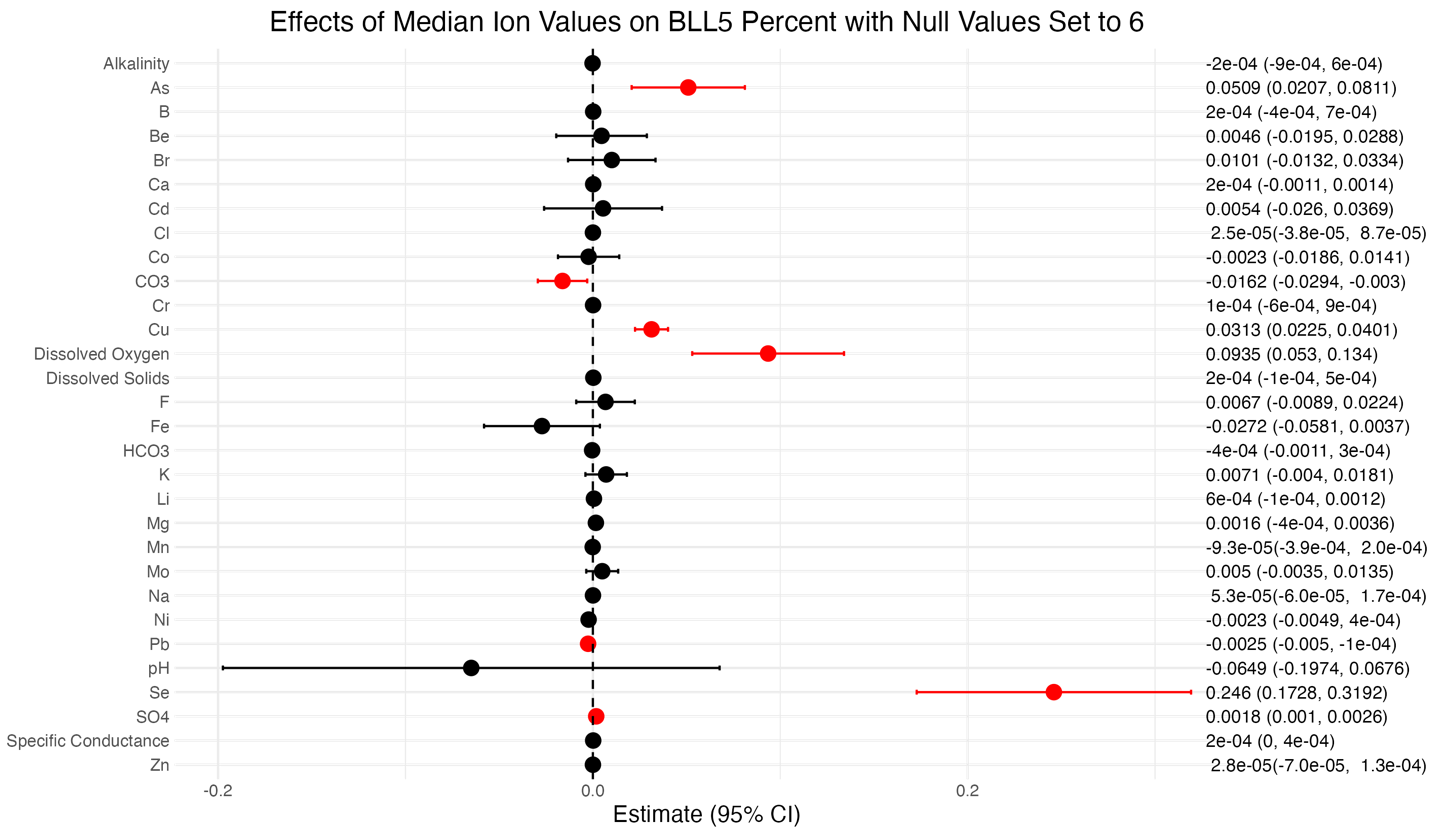


Figure S6: Association between one unit increase in median of groundwater chemicals and change in % of children with elevated blood lead levels above 5 µg/dl with NA values of BLL5 set to 6. The significance level was determined using a p-value less than 0.05.


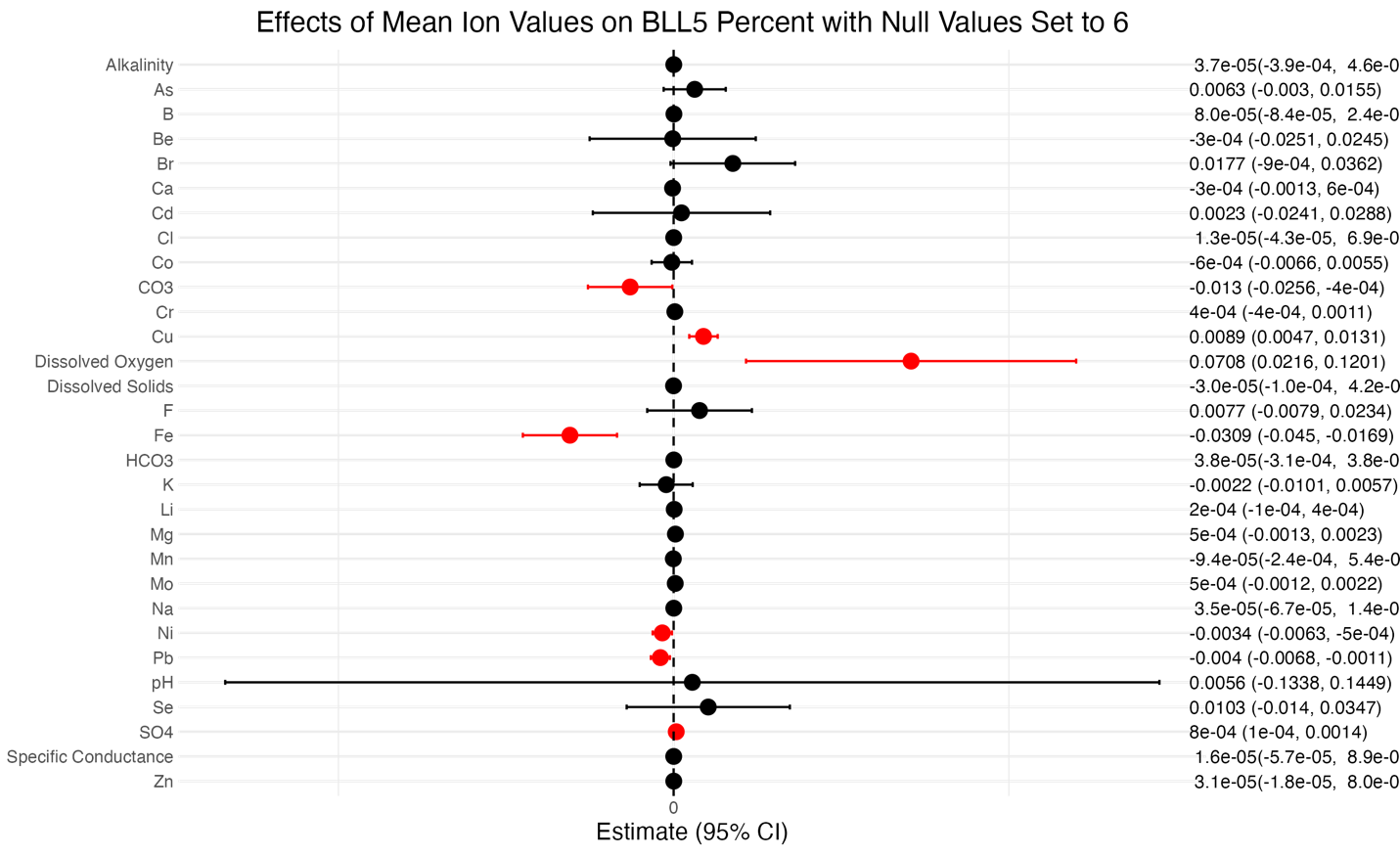


Figure S7: Association between one unit increase in mean of groundwater chemicals and change in % of children with elevated blood lead levels above 5 µg/dl with NA values of BLL5 set to 6. The significance level was determined using a p-value less than 0.05.


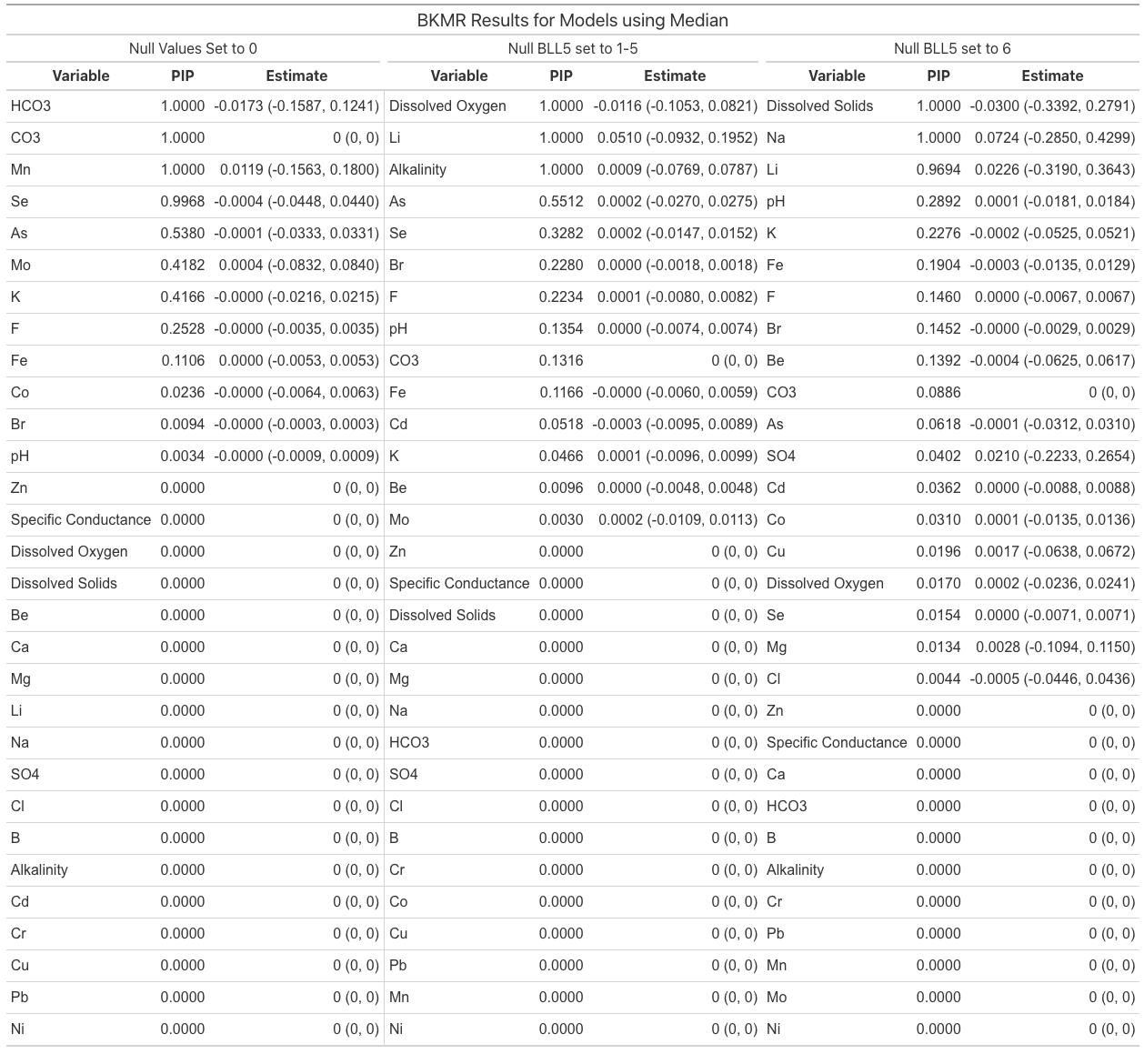


Table S2: Results of BKMR using median values of groundwater chemicals when setting null BLL5 to 0, randomly to 1-5, and 6.


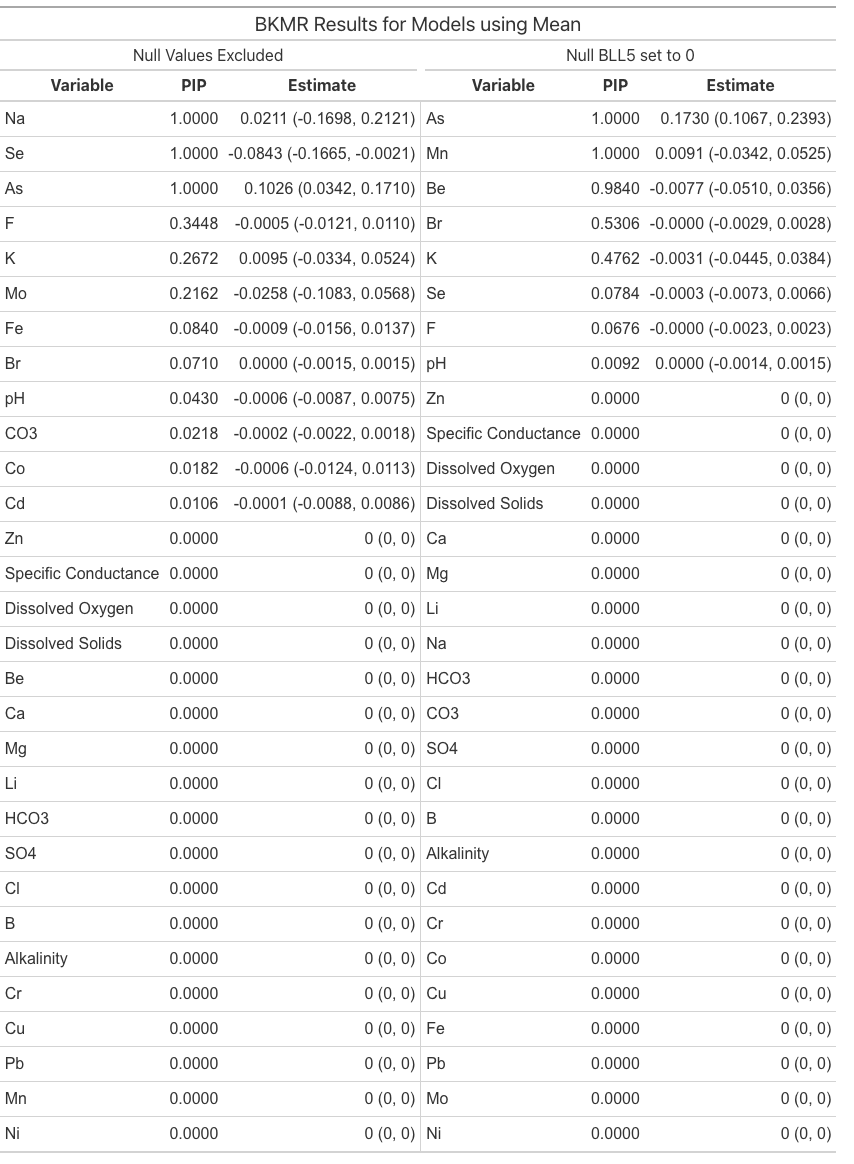


Table S3: Results of BKMR using mean values of groundwater chemicals when null values of BLL5 were excluded and set to 0.

Table S4: Results of BKMR using mean values of groundwater chemicals when setting null BLL5 randomly to 1-5 and to 6.


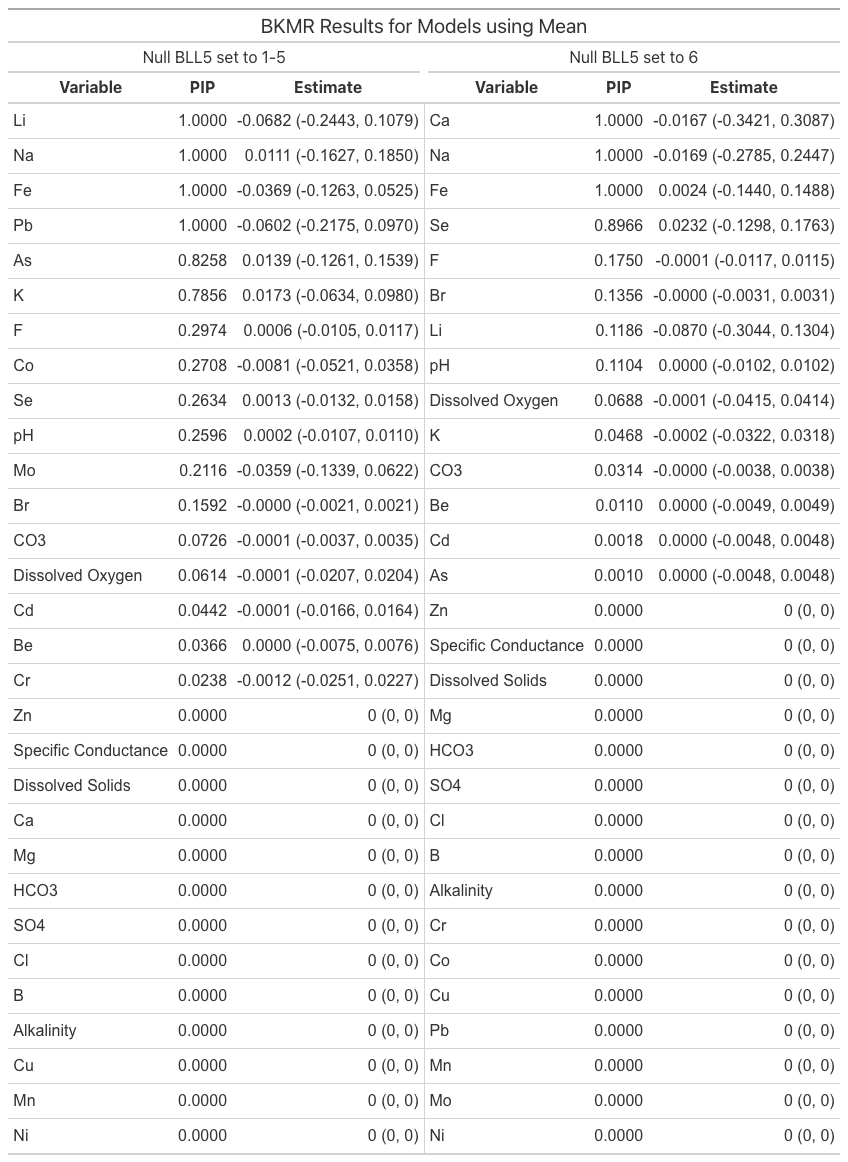

Supplement: Supplementary file 1 — Supporting Information S1 [file GH2-10-e2025GH001670-s001.docx]
